# A Cross-Layer Framework Integrating RF and OWC with Dynamic Modulation Scheme Selection for 6G Networks

**DOI:** 10.3390/s26030926

**Published:** 2026-02-01

**Authors:** Ahmed Waheed, Borja Genoves Guzman, Somayeh Mohammady, Maite Brandt-Pearce

**Affiliations:** 1 School of Electrical and Electronics Engineering, Technological University Dublin (TUD), D07 H6K8 Dublin, Ireland; somayeh.mohammady@tudublin.ie; 2 Departamento Teoría de la Señal y Comunicaciones, Universidad Carlos III de Madrid, Av. de la Universidad 30, 28911 Leganés, Spain; bgenoves@ing.uc3m.es; 3 Department of Electrical and Computer Engineering, University of Virginia, Charlottesville, VA 22904, USA; mb-p@virginia.edu

**Keywords:** cross-layer framework, hybrid RF/OWC networks, modulation schemes, OFDM, OTFS, OCDM, SCMA, OFDM-IM, 6G

## Abstract

With the rapid evolution of wireless networks, the need to explore novel technologies to meet the demands of future systems, particularly 6G, has become a significant challenge. One promising solution is integrating radio frequency (RF) and optical wireless communication (OWC) technologies to leverage their unique strengths. This paper introduces a novel model for integrating RF and OWC technologies within the framework of emerging 6G. The main objective of this approach is the dynamic technology selection (TS) and modulation scheme selection (MSS), which play a pivotal role in optimizing network efficiency and adapting to diverse 6G requirements. The proposed cross-layer architecture integrates the application layer, network layer based on a software-defined network (SDN), and physical layer consisting of a hybrid cell and software-defined radio with optical functionality (SDR-O). This approach facilitates real-time decision-making based on environmental factors and application requirements.

## 1. Introduction

The evolution toward 6G networks is driven by the growing demands of emerging applications such as digital twins, augmented reality, virtual reality, ultra-dense connectivity, vehicle-to-vehicle communication, advanced intelligent systems, and coordinated sensing, among others [1]. The key requirement in 6G is meeting the diverse needs of various services, each with its specific performance priorities. These include factors like the efficient use of spectrum, high data rates, low latency, and energy efficiency, which differ depending on the application. For instance, massive Internet of Things (IoT) focuses on low power consumption and efficient spectrum use, while autonomous vehicles require extremely low latency and high reliability. These advanced applications demand performance levels beyond those of 5G networks.

Existing radio frequency (RF) technologies have been the backbone of wireless communication to support the requirements of planned 6G applications. However, the number of connected devices is expected to increase by up to 30 billion by 2030. This rapid growth augments the congestion and interference in the RF spectrum, which severely limits the ability of the communication systems to deliver high data rates and consistent performance.

Optical wireless communication (OWC) utilizes a wide spectrum of frequencies, providing abundant bandwidth able to deliver high data rates and ultra-low latency. The advantage of RF technology to cover long distances, integrating with the high-capacity feature of OWC, offers a great solution that has the potential to meet the requirements of diverse 6G applications [2]. For instance, in the smart cities scenario, RF technologies can provide extensive citywide connectivity, while OWC can be deployed in high-density hotspots like stadiums, where a high data rate is essential [3]. One of the key challenges with this integration is performing an appropriate technology selection (TS) and a modulation scheme selection (MSS) based on the environment and application [4].

**Figure 1:**
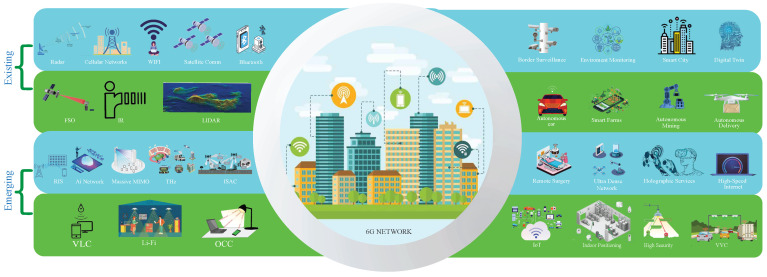
6G network in every aspect of life. The left column presents existing and emerging RF (blue) and OWC (green) technologies. The right column illustrates their corresponding target applications in 6G.

Our vision for 6G networks and beyond is to take advantage of RF and OWC in a single framework, enabling seamless and high-performance communication for various applications as shown in Figure 1. While substantial research exists on the convergence of RF and fiber-based systems [5,6,7], there is a lack of literature addressing the integration of RF and OWC technologies, particularly through the dynamic management of emerging modulation schemes. The main contribution of this paper is proposing an innovative approach to integrating RF and OWC systems within a cross-layer framework that includes the application, network, and physical layers.

## 2. Existing RF and OWC Technologies

An abundance of research has been done in each of the domains, RF and OWC. In the following, existing and emerging RF and OWC technologies are summarized and represented in Figure 1, where important applications of both technologies in 6G are also considered. For example, the left column includes representative technologies such as Reconfigurable Intelligent Surfaces (RIS) and Light-Fidelity (Li-Fi), while the right column illustrates target applications like autonomous driving and holographic communication.

### 2.1. RF Technologies

RF technology is known to be an ideal option for long-range line-of-sight (LOS) and non-line-of-sight (NLOS) communication, especially in multipath propagation scenarios. It is reliable for mobile and portable use. The key existing and emerging RF technologies are:

#### 2.1.1. Existing

*Cellular networks*, such as 5G New Radio, offer high-speed, low-latency, and long-range wireless connectivity. This technology is ideal for real-time applications and smart cities, but it requires dense infrastructure when using high-frequency bands. *Wi-Fi* provides high-speed communication with both LOS and NLOS capabilities [3]. It is cost-effective to deploy and works with a wide range of devices, making it a great option for medium-range communication. *Bluetooth* is a short-range and low-power RF technology that is ideal for wireless peripherals and IoT devices. *Satellite communication* offers global coverage, especially in remote areas where terrestrial networks are unavailable, but it has a high latency and cost. Recent developments in low Earth orbit (LEO) are improving performance and minimizing delays. *Radar communication* is designed for detection and tracking, making it ideal for environment monitoring and border surveillance applications.

#### 2.1.2. Emerging

*THz communication* exploits higher frequencies for wireless communication, providing a much larger bandwidth. The development of new equipment and antennas is required to support THz transmission. *Reconfigurable intelligent surfaces (RIS)* are used to manipulate electromagnetic waves to improve signal strength and coverage in challenging environments. To integrate this technology into existing networks, smart surfaces, sensors, and controllers will need to be installed to manage their functionalities in real time. *Integrated sensing and communication (ISAC)* combines communication and sensing functionalities into a single network, enabling new use cases such as autonomous vehicles, smart cities, and industrial automation. *Massive MIMO (Multiple Input Multiple Output)* uses a large number of antennas to increase the capacity and efficiency of wireless networks. The current infrastructure will need to be upgraded with advanced signal processing techniques and new hardware to implement this technology. *Artificial intelligence (AI) and machine learning (ML)-based networks* are used to optimize network performance. The integration of AI into existing infrastructure will require advanced software and intelligent systems capable of managing complex tasks and real-time decision-making.

### 2.2. OWC Technologies

OWC is ideal for high-speed and LOS applications. It provides high data rates, enhanced security, and immunity to RF interference, making it suitable for dense environments, secure communications, and indoor systems. However, OWC is more vulnerable to weather conditions and requires clear, unobstructed paths to function reliably. The key existing and emerging OWC technologies are:

#### 2.2.1. Existing

*Infrared communication (IR)* using LEDs offers low power consumption for short-range communication in unobstructed environments. It is commonly used in remote control systems, laptops, and smartphones. *Free-space optics (FSO)* typically uses a laser beam to transmit data in outdoor point-to-point long–range communications. This offers high-speed, secure data transmission with low latency and is suitable for autonomous delivery. *Light detection and ranging (LiDAR)* technology utilizes infrared laser light to measure distances and is mainly used for sensing and mapping. It is an ideal technology for autonomous vehicles, smart farms, and autonomous mining.

#### 2.2.2. Emerging

*Light-Fidelity (Li-Fi)* is an alternative to traditional Wi-Fi that, instead of RF signals, uses the visible light spectrum, as well as ultraviolet (UV) and IR light, to transmit data. This enables Li-Fi to offer several advantages, including significantly higher data transmission rates, enhanced security due to its confined line-of-sight operation, and reduced interference, making it particularly suitable for environments where RF communication is limited or undesirable, such as hospitals, aircraft cabins, or industrial settings. However, Li-Fi requires LOS between transmitter and receiver, which limits its operation to short ranges [8,9]. *Visible light communication (VLC)* is the physical layer of Li-Fi when it uses visible light. It offers a unique dual-use capability by integrating data transmission with architectural LED lighting. This helps reduce energy consumption and infrastructure costs, offers high-speed communications, and is immune to RF interference, making it ideal for indoor positioning and secure communications. *Optical camera communication (OCC)* modulates light transmitted by an LED array or screen and receives it by using a camera. The data is encoded into light patterns, which can be detected and decoded by the camera’s sensor. This is ideal for very low data rate, high-security applications.

## 3. Key 6G Modulation Schemes

MSS directly impacts metrics such as spectral efficiency, power efficiency, etc., which are summarized in Table 1. In this paper, we refer to spectral efficiency as the Shannon capacity over a given bandwidth, and to power efficiency as the required signal-to-noise ratio (SNR) for a given bit error rate (BER). In the following, we detail emerging 6G RF and optical modulation schemes.

### 3.1. RF Modulation Schemes

Traditionally, single-carrier waveforms, including Quadrature Amplitude Modulation (QAM) and Phase Shift Keying (PSK), are fundamental modulation schemes in mobile communication systems. However, when operating at high speed, these modulation schemes are more susceptible to frequency-selective fading in environments with significant multipath propagation. To address these challenges, multi-carrier modulation (MCM) schemes have gained significant interest [10]. In these transmission schemes, the available channel bandwidth is divided into multiple sub-channels, known as subcarriers, to avoid frequency selectivity. In MCM, multiplexing between users can be performed in both the time and frequency domains. In the following, we detail the most important RF MCM schemes that are candidates for 6G.

#### 3.1.1. Orthogonal Frequency Division Multiplexing (OFDM)

OFDM is the most popular MCM [11]. In this modulation scheme (MS), subcarriers are precisely arranged to be orthogonal. These modulated subcarriers are converted from the frequency domain to the time domain by using the inverse fast Fourier transform (IFFT). To improve robustness against inter-symbol interference (ISI) due to multiple paths, a cyclic prefix (CP) is added to each symbol. OFDM systems allow for a one-tap equalizer structure at the receiver. OFDM is widely used in 4G and 5G due to these flexibilities and its efficiency in handling high data rates. It is the ideal choice for 6G applications requiring a high data rate, like augmented and virtual reality (AR/VR). The main challenges associated with OFDM are a high peak-to-average-power ratio (PAPR), sensitivity to frequency and timing offsets, and CP overhead, making it unsuitable for 6G applications that require low power and high mobility.

#### 3.1.2. Orthogonal Time Frequency Space (OTFS)

The OTFS scheme embeds the QAM information symbols into the delay-Doppler (DD) domain [12], achieving a more robust representation of rapidly time-varying channels through DD domain mapping. This approach reduces degradation caused by frequency and time selectivity in high-mobility applications. The information is then transformed into the time-frequency (TF) domain with the help of the inverse symplectic finite Fourier transform (ISFFT). Using the Heisenberg transform, the TF symbols are mapped to the time domain. The process is reversed at the receiver to retrieve the original data. OTFS mitigates the effects of inter-carrier interference due to phase noise and shows resilience to narrow-band interference. However, OTFS has a high complexity and computational cost because it involves a two-dimensional mapping of data to the time-frequency domain.

#### 3.1.3. Orthogonal Chirp Division Multiplexing (OCDM)

In this scheme, multiple chirped waveforms are orthogonally multiplexed to achieve the highest possible communication rate [13]. Each chirp signal’s amplitude and phase are modulated by selecting the appropriate QAM or PSK modulation scheme. The OCDM signal is produced using the inverse Discrete Fresnel transform (DFnT). OCDM offers better performance in frequency-selective and/or time-selective channels. OCDM’s ability to decompose complex radar signals into smaller chirp-based sub-blocks enhances the overall system performance, making it well-suited for radar 6G applications. However, OCDM has moderate complexity in code generation, challenges with interference sensitivity, and medium spectrum efficiency in large-scale systems.

#### 3.1.4. Sparse Code Multiple Access (SCMA)

6G networks will confront significant challenges from having to serve a vast number of concurrent applications with ultra-dense connections [14]. SCMA offers a novel nonorthogonal multiple access scheme for massive connectivity and ultra-low latency. In this scheme, multiple users share the same frequency and time resources simultaneously, and each user is assigned a unique codebook, which is a predefined set of multidimensional code words. A message-passing algorithm detector is used to exploit the codebook’s sparsity, which helps mitigate inter-user interference. SCMA enhances the overall system capacity by enabling different users to share the same resources. However, SCMA faces challenges, such as complexity of codebook design and sensitivity to channel conditions, impacting its robustness and efficiency in dynamic environments.

#### 3.1.5. Index Modulation (IM)

In recent years, IM schemes have gained significant interest for 6G networks due to their low power consumption and robustness against interference. OFDM with index modulation (OFDM-IM) is an IM modulation scheme that utilizes the indices of active subcarriers to transmit additional information [15], without requiring additional bandwidth [16]. In this scheme, only a subset of subcarriers in each symbol is activated at any given time instead of all the subcarriers, which reduces the power consumption of the transmitted signal, making this modulation scheme ideal for IoT applications. To decode the information correctly, a complex detector is required at the receiver, which increases the computational complexity.

### 3.2. Optical 6G Modulation Schemes

Information in OWC is embedded into the light signal for transmission and reception by using coherent or noncoherent methods. Due to the dynamic environment of 6G applications, this paper focuses on emerging 6G optical noncoherent modulation schemes, which are based on intensity modulation and direct detection (IM/DD), as they offer a simpler, more robust, and cost-effective solution. Additionally, IM/DD is less sensitive to rapid changes in channel conditions and can better support the high-speed, low-latency requirements expected in 6G systems.

#### 3.2.1. Optical-OFDM (O-OFDM)

Applying OFDM to OWC requires making the OFDM transmitted signal real and nonnegative [17]. First, a real signal is obtained after the IFFT by introducing Hermitian symmetry into the QAM symbols, i.e., the second half of subcarriers is a Hermitian-symmetric copy of the first half of the subcarriers, which halves the spectral efficiency. Second, the signal is converted into a positive signal, which can be accomplished mainly by two methods: (1) Introducing a DC bias to the signal to shift it entirely into the positive domain, which leads to the so-called direct current-biased O-OFDM (DCO-OFDM); (2) Using only the odd subcarriers and then clipping the time-domain signal at zero, removing all the negative values, which is the so-called asymmetrically clipped O-OFDM (ACO-OFDM). DCO-OFDM may offer a larger data transmission as a result of effectively using more subcarriers, whereas ACO-OFDM may save energy by not requiring a DC value.

#### 3.2.2. Optical-OTFS (O-OTFS)

In O-OTFS, information symbols are mapped to a two-dimensional grid in the delay-Doppler domain as summarized in Section 3.1. Similar to traditional O-OFDM, O-OTFS employs Hermitian symmetry, and either a DC offset or asymmetric clipping to produce a positive-valued waveform [18]. In contrast to O-OFDM, where each symbol requires a CP per symbol to remove ISI, O-OTFS requires only a single CP for the entire frame. This approach significantly reduces overhead and improves the bandwidth efficiency of the system, which is crucial for high-speed optical communication applications.

#### 3.2.3. Optical-OCDM (O-OCDM)

It should be noted that a nonnegative valued signal is obtained by applying the DC biasing or asymmetric clipping technique to the OCDM waveform before transmitting it into the optical wireless channel. The receiver, after removing the DC bias or asymmetric clipping, demodulates the signal by using the DFnT.

#### 3.2.4. Optical-SCMA (O-SCMA)

O-SCMA is a nonorthogonal multiple access scheme similar to the RF version, SCMA, but the sparse code words are real as needed for intensity modulation. The integration of SCMA with OWC is suitable for many 6G applications, such as high-density communication, shopping centres, and crowded stadiums.

#### 3.2.5. Optical-IM (O-IM)

The implementation of OFDM-IM in OWC systems (O-OFDM-IM) presents a compelling and innovative design challenge. The difference on the transmitter side is that Hermitian symmetry is applied after subcarrier mapping to ensure the signal is real, in addition to a DC biasing or asymmetrical clipping after the IFFT.

### 3.3. Performance Comparison

In this section, we evaluate the performance of different RF and OWC modulation schemes in AWGN channel conditions. The code to obtain these results is publicly available at (https://github.com/Ahmedwaheed4181/RF-and-Optical-Modulation-Scheme-Comparison.git (accessed on 1 July 2025)). The simulations are conducted using QPSK modulation with 32 subcarriers. In OFDM-IM, each group consists of 8 subcarriers, of which 4 subcarriers are active per group. Three metrics are used to evaluate the performance of all modulation schemes, namely BER, PAPR, and spectral efficiency. Simulations of the optical modulation schemes have been performed based on both DCO and ACO classifications. However, the results presented include only the best-performing optical OFDM variant for each scenario, indicated by the corresponding “DCO” or “ACO” class. This approach is adopted to avoid redundancy and to improve clarity, as presenting multiple sub-optimal variants would not provide additional insight into system performance comparisons.

**Figure 2:**
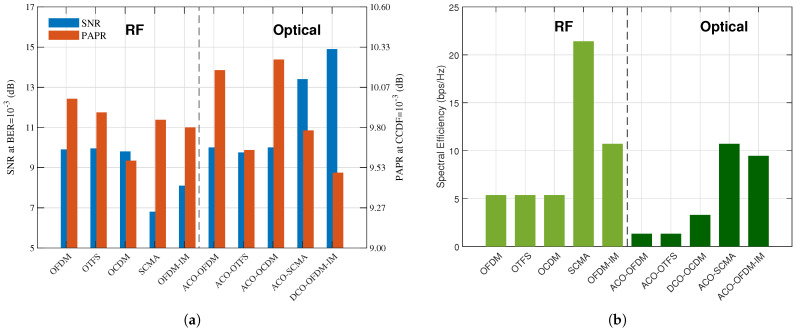
Performance analysis of RF and optical modulation schemes with no Multipath. (**a**) Performance analysis of SNR (blue bars) vs. PAPR (orange bars) for RF and optical modulation schemes. (**b**) Performance analysis of spectral efficiency for RF (light green) and optical (dark green) modulation schemes.

Figure 2a presents a comparative analysis using dual-axis bar plots, illustrating the required SNR at a target BER = $10^{-3}$ and the PAPR evaluated at a complementary cumulative distribution function (CCDF) = $10^{-3}$. Let us first analyze the SNR results. OFDM/ACO-OFDM, OTFS/ACO-OTFS, and OCDM/ACO-OCDM require practically the same SNR for our target BER, which is larger than the one required by SCMA and OFDM-IM in RF, and lower than the one required by ACO-SCMA and DCO-OFDM-IM in OWC. The DC bias in DCO-OFDM-IM increases noise and, consequently, increases the required SNR. The index modulation (IM) component is sensitive to the added noise because detecting active subcarriers relies on energy detection. Noise can lead to incorrect detection of active subcarriers, causing errors in both index bits and data bits. Differently, ACO-OFDM-IM clips negatives, which introduces distortion. Unlike conventional ACO-OFDM, the IM component spreads information across subcarriers, making it vulnerable to clipping noise. SCMA has the lowest required SNR because it uses complex-valued codebooks, offering more degrees of freedom. The detector can distinguish user signals clearly in the complex domain. On the other hand, in ACO-SCMA, making the codebook real-valued and non-negative reduces the Euclidean distance between code words, making them harder to distinguish at the receiver and then increasing the required SNR. The clipping of negatives further distorts the signal, which contributes to increasing the required SNR for a given BER.

We now analyze the PAPR for a CCDF = $10^{-3}$ also depicted in Figure 2a. The PAPR of most schemes falls within the range of 9.0–10.5 dB, with OFDM and OTFS showing slightly higher values, while SCMA and OFDM-IM exhibit slightly lower values due to their sparsity. This is due to their shared IFFT-based structure and QPSK modulation. OFDM and OTFS show the highest PAPR, around 9.9 dB, from subcarrier superposition, while OFDM-IM and SCMA achieve a marginal reduction through sparsity, around 9.8 dB. Their optical variants (DCO/ACO) balance clipping and biasing effects.

Figure 2b shows the spectral efficiency achieved by each scheme. OFDM/OTFS/OCDM achieve baseline spectral efficiency (5.35 bps/Hz), while OFDM-IM (10.7 bps/Hz) and SCMA (21.4 bps/Hz) gain through index modulation and non-orthogonal multiplexing, respectively. Optical variants exhibit trade-offs: ACO schemes suffer severe spectral efficiency loss from a quarter subcarrier utilization, except ACO-OFDM-IM (9.45 bps/Hz), which combines index modulation with efficient clipping. DCO-OCDM (3.82 bps/Hz) outperforms ACO.

**Figure 3:**
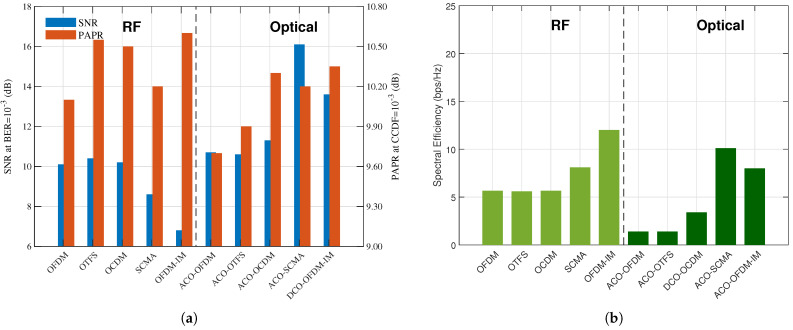
Performance analysis of RF and optical modulation schemes with Multipath. (**a**) Performance analysis of SNR (blue bars) vs. PAPR (orange bars) for RF and optical modulation schemes. (**b**) Performance analysis of spectral efficiency for RF (light green) and optical (dark green) modulation schemes.

To evaluate robustness under more realistic impairments, we extend our simulations to multipath channel analysis. The multipath model consists of three taps with gains [0.8, 0.05, 0.1], representing a frequency-selective channel with a strong main path and two weaker multipath components. This provides insight into how different modulation schemes perform under realistic frequency-selective fading conditions, which is crucial for dynamic TS/MSS decisions in varying environments. The performance comparison with multipath is illustrated in Figure 3.

The performance comparison under multipath conditions demonstrates significant differences from the no multipath conditions. SCMA exhibiting superior spectral efficiency in AWGN (21.4 bps/Hz), suffers a 60% reduction under multipath (8.1 bps/Hz) due to codebook sensitivity to channel estimation errors. On the other hand, OFDM-IM demonstrates improved robustness, with SNR requirements decreasing from 8.1 dB to 6.8 dB, benefiting from frequency diversity in selective fading channels. Optical variants show distinct behaviors: ACO-SCMA degrades severely (SNR increases from 13.4 dB to 16.1 dB), while DCO-OFDM-IM shows moderate improvement.

The performance comparison of RF and optical MSs directly supports our paper’s central thesis that no single modulation scheme can fully meet the diverse requirements of future 6G networks. This is why the system design should incorporate multiple modulation schemes within a single platform, supporting both RF and optical functionalities. To address this challenge, we propose in the next section a hybrid framework that dynamically combines RF and optical MSs based on application demands.

## 4. Proposed Cross-Layer Hybrid Approach

Our proposed cross-layer hybrid approach seamlessly integrates the application, network, and physical layers to support adaptive communication in 6G networks. Its primary goal is to choose the most suitable RF and OWC technologies based on environmental conditions and application needs, and then dynamically select key RF or Optical 6G modulation schemes, as outlined in Section 3.

### 4.1. Application Layer

This layer addresses the specific requirements of diverse 6G use cases by mapping application demands to their respective key performance indicators (KPIs). These KPIs drive the translation of user demands into network policies. The KPIs identified for 6G systems include:

1. Ultra-high data rate.
2. Ultra-low latency.
3. Massive connectivity.
4. High mobility.
5. High-precision positioning.
6. Ultra reliability.
7. Low power consumption/High energy efficiency.

These KPIs serve as a critical tool for evaluating and optimizing network configurations. By mapping 6G applications to their corresponding KPIs, the network can dynamically adapt to the specific requirements of each application, ensuring optimal performance. Table 2 provides an overview of key applications, their requirements, and their associated KPIs.

### 4.2. Network Layer

**Figure 4:**
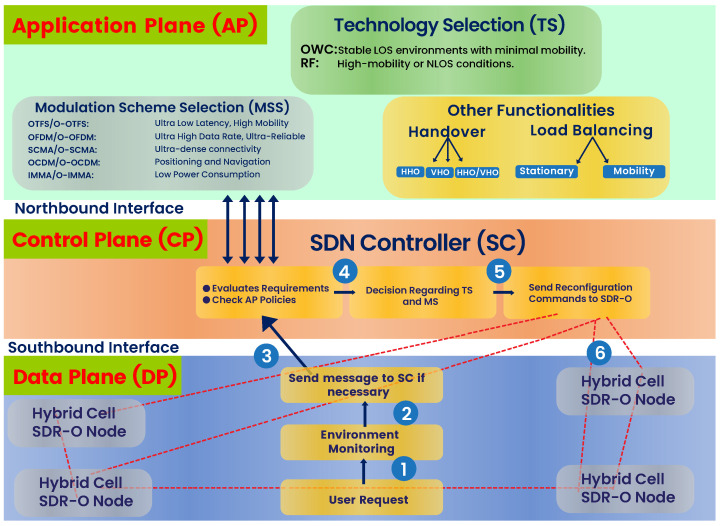
Network layer of the proposed cross-layer hybrid approach for 6G networks.

The real-time MSS and TS based on the environment and application demands are challenging tasks. However, software-defined networks (SDNs) have emerged as a solution to manage and optimize the operation of modern networks. SDN consists of three planes represented in Figure 4.

*Application Plane (A-Plane):* This plane is responsible for translating the 6G application requirements into policies, including those related to TS and MSS, and other functionality such as handover and load balancing. There are two types of handover to ensure seamless connectivity when the user moves between different network cells or technologies. The first is vertical handover (VHO) between two different technologies (RF to/from OWC). The second is horizontal handover (HHO) between cells within the same technology. Load balancing in this hybrid network ensures the dynamic allocation of network traffic across RF and OWC links to ensure optimal performance, reliability, and resource utilization. Our proposed approach considers two main scenarios of load balancing: stationary and mobile users. For stationary users, the focus is on efficiently distributing traffic between RF and OWC to optimize resource utilization and performance. In contrast, for mobile users, the priority is ensuring seamless connectivity and optimal performance despite rapid changes in channel conditions as the user moves.

*Control Plane (C-Plane):* The core component of this cross-layer hybrid approach is the SDN controller (SC) living in the C-plane, which manages the network resources, policy enforcement, and dynamically adapts configurations based on real-time requirements. The user demand from the physical layer via the southbound interface is received by the SDN controller. The C-Plane evaluates user requirements and reviews the policies set by the A-Plane via the northbound interface. Based on these policies, it enforces decisions for MSS and TS and then sends reconfiguration commands to the data plane (D-Plane).

*Data Plane (D-Plane):* The D-Plane is responsible for the actual routing or filtering of data packets within the network. This plane executes the reconfiguration commands received from the SC. It operates at a low level, ensuring efficient and reliable data flow while adhering to the rules and policies defined by the A-Plane and enforced by the C-Plane.

As illustrated in Figure 4, the operation of the whole network layer, including the A-Plane, C-Plane, and D-Plane, is described in the following steps:

*Step* *1:* A request is received by the A-Plane from the users or requirements from the applications, such as demands for high-speed communication, low latency, or reliable connectivity.
*Step* *2:* The system actively monitors environment conditions, including LOS, mobility, and network load, to identify the best technology, RF or OWC, in the D-Plane.
*Step* *3:* Based on the environmental monitoring results, the system sends information to the SC in the C-Plane. The SC evaluates the requirements and verifies these with the policies defined in the A-Plane.
*Step* *4:* The SC decides on the appropriate modulation scheme and technology based on the A-Plane to meet the demand from users or applications.
*Step* *5:* Finally, the SC sends the reconfiguration commands to the software-defined radio with optical functionality (SDR-O) node in the D-Plane to implement the setting for efficient and seamless communication.

### 4.3. Physical Layer

This layer is responsible for the transmission and reception of data over the wireless (RF or optical) channel.

The combination of software-defined radio (SDR) flexibility with optical communication strengths promises an innovative solution for future 6G networks. The SDR equipped with RF antennas and optical transceivers, named here SDR-O, enables real-time MSS and TS, allowing it to adapt to various network environments and user requirements. The main advantage of SDR-O in this approach is that it can adapt to various wireless communication standards, RF and optical, by simply changing the software. This layer is also responsible for implementing the modulation scheme to adapt the data signal as per the user and environmental requirements for transmission over the wireless medium. This layer also provides real-time feedback to the Network Layer on link quality.

### 4.4. Decision Metrics and Real-Time Operation

To enable dynamic TS and MSS, the SDN controller operates on a closed-loop system driven by real-time metrics from the physical and network layers.

#### 4.4.1. Decision Inputs and Handover Logic

The controller continuously monitors a set of key performance and environmental indicators:

- Channel State Information (CSI): SNR, delay spread, and Doppler shift.
- Link Quality Indicators: BER, packet loss rate, Received Signal Strength Indicator (RSSI) for RF, and received optical power for OWC.
- Network Load: Traffic volume per link and queueing delay.
- Environmental Conditions: LOS or Non-LOS status (detected via camera/LiDAR sensors for OWC), user mobility speed, and weather data for outdoor OWC links.
- Application KPIs: Latency, throughput, and reliability targets as defined in Table 2.

Vertical Handover (VHO) and Horizontal Handover (HHO) are triggered using a weighted scoring model [19]. When the composite score of the current link falls below a threshold, or a superior link becomes available, the controller initiates a handover. Load balancing for stationary users allocates traffic based on real-time link capacity, while for mobile users, it employs predictive handover based on user trajectory.

#### 4.4.2. Latency Bounds for Real-Time Reconfiguration

The feasibility of this dynamic operation depends on stringent latency bounds for each step in the control loop. The estimated timescales based on state-of-the-art SDN and SDR platforms are summarized in Table 3. This end-to-end process ensures that optimal performance is defined and maintained according to the application’s KPIs mentioned in Table 2. The framework then continuously adjusts to changes in the channel and network, ensuring these targets are met in real time.

### 4.5. Illustrative Example of the Proposed Cross-Layer Framework

**Figure 5:**
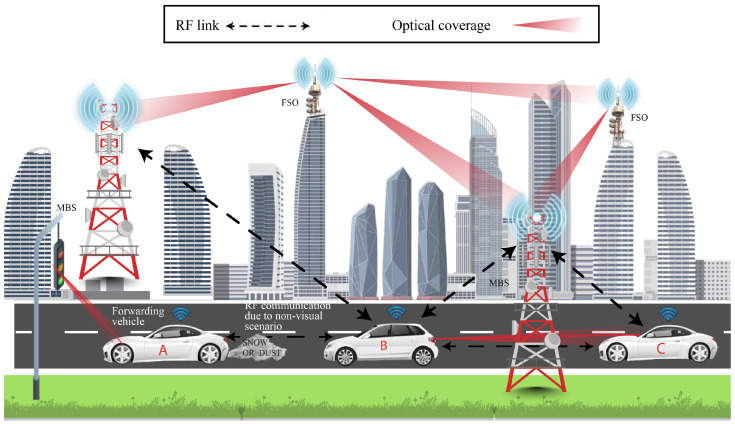
Illustrative example of the proposed scheme.

Figure 5 illustrates an example of the implementation of the proposed cross-layer hybrid communication scheme. In this vehicle-to-vehicle communication scenario, the Application layer maps user demands, such as high-speed connectivity, low latency, and high reliability, into KPIs. These KPIs are then translated into network policies that guide the decision-making process. The Network layer managed by the SC receives demands from the Application layer. The SC evaluates the user requirements and cross-references them with the policies defined in the A-Plane to determine the most suitable modulation scheme and technology for the current scenario.

The condition is identified by the D-Plane in the Network layer as LOS between Vehicle B and Vehicle C, making OWC the ideal choice for high-speed data transmission. However, the condition between Vehicle A and Vehicle B is classified as NLOS due to obstructions necessitating the use of RF communication to maintain connectivity. As vehicles are highly mobile, the Network layer of our proposed system selects OTFS as the optimal modulation scheme for both RF and OWC communication. Finally, the SC sends reconfiguration commands to the SDR-O in the physical layer for transmission. This adaptive mechanism ensures seamless communication, even in dynamically changing environments, and exemplifies the flexibility and efficiency of the proposed cross-layer hybrid approach.

## 5. Conclusions and Remaining Challenges

This paper presents an innovative cross-layer hybrid approach integrating RF and OWC technologies to address the diverse and demanding requirements of 6G networks. By leveraging dynamic MSS with a comprehensive framework that includes the application layer, network layer based on SDN, and physical layer consisting of a hybrid cell and SDR-O, we provide a flexible and scalable solution for future wireless networks.

However, there are still several challenges that need further research to unlock the potential of cross-layer hybrid networks, such as: developing advanced algorithms for efficient spectrum sharing and resource allocation to maximize the benefits of hybrid RF/OWC technologies; extending security frameworks across all layers to mitigate vulnerabilities; optimizing the power use of hybrid transceivers to ensure energy efficiency while managing the high-energy demands of real-time network adjustments; developing standard protocols to ensure interoperability between diverse technologies, devices, and vendors in hybrid networks; and implementation of advanced technologies such as AI and ML into the cross-layer framework to predict and adapt to dynamic conditions. Addressing these challenges will enable the development of optimized, reliable, and scalable cross-layer hybrid networks, progress that is crucial for meeting the demands of 6G communication systems.

## Figures and Tables

**Table 1:** Comparison of emerging 6G RF and optical modulation schemes. When features of RF and optical modulation schemes differ, the optical feature is written in parentheses.

Parameters	(O-)OFDM	(O-)OTFS	(O-)OCDM	(O-)SCMA	(O-)OFDM-IM
Spectral Efficiency	Low	Low	Medium	High (Medium)	Medium
Power Efficiency	Low	Low	Low	High (Low)	High (Low)
Orthogonality	Yes	Yes	Yes	No	Yes
Cyclic Prefix	Yes	Optional	Optional	No	Yes
Complexity	Low	High	Medium	High	Medium
CFO Resiliency	Low	High	High	Medium	Low
Flexibility	High	Medium	Medium	Low	High
Support Mobility	Low	High	Medium	Medium	Low
Applications	High data rate	High mobility	Positioning and sensing	Massive connectivity	Low power consumption

**Table 2:** The 6G key applications with target KPIs.

6G Key Applications	KPI Numbers	Targets
Vehicle-to-vehicle communication, Vehicle-to-infrastructure communication, Automatic drive	2, 4	Ultra-low latency: sub-second (<1 ms), High mobility: up to 1000 km/h
Ultra-high definition video, Holographic services (Augmented reality, Virtual reality)	1, 6	Peak data rate up to 1 Tbps, User experiences: 1–10 Gbps, Reliability: 99.99999%
Super dense population (Crowded shopping malls, Football stadiums)	3	Massive connection: up to $10^{8}$ devices/km^2^
Positioning and navigation, Indoor/Outdoor precise positioning	5	Outdoor: meter level, Indoor: centimeter level
Internet of bio-nano-things, Healthcare wearables, implantable sensors	7	Micro/Nano-watt-level power consumption for passive sensors

**Table 3:** Estimated latency breakdown for dynamic modulation scheme selection (MSS) and technology selection (TS).

Process Step	Estimated Timescale	Description
Environment Reporting	1–10 ms	Collection of CSI, link quality indicators, and LOS/NLOS status from physical-layer sensors.
SDN Controller Decision	5–20 ms	Policy matching, MSS/TS algorithm execution, and handover decision based on real-time inputs.
SDR-O Waveform Switching	<5 ms	Reconfiguration of the RF/optical front-end and modulation parameters in the SDR-O.
Total Reconfiguration Latency	∼10–35 ms	End-to-end latency supports dynamic adaptation for 6G applications with latency budgets ≥ 50 ms.

## Data Availability

The MATLAB code supporting the findings of this study is available in the GitHub repository at https://github.com/Ahmedwaheed4181/RF-and-Optical-Modulation-Scheme-Comparison.git (accessed on 1 July 2025). The specific link is provided in the article.
